# A case report of severe recurrent varicella in an ankylosing spondylitis patient treated with adalimumab – a new side effect after 15 years of usage

**DOI:** 10.1186/s12879-019-3768-y

**Published:** 2019-02-07

**Authors:** Tomislava Skuhala, Anita Atelj, Jelena Prepolec, Mahmoud Al-Mufleh, Andrija Stanimirović, Dalibor Vukelić

**Affiliations:** 1 University Hospital for Infectious Diseases “Dr. Fran Mihaljević”, Mirogojska 8, 10000 Zagreb, Croatia; 2 School of Dental Medicine, University of Zagreb, Zagreb, Croatia; 3 School of Medicine, University of Zagreb, Zagreb, Croatia; 4 Resident in Infectious Diseases, County Hospital Čakovec, Čakovec, Croatia; 5 Department of Clinical Medicine, University of Applied Health Sciences, Zagreb, Croatia

**Keywords:** Adalimumab, Varicella virus, Severe recurrent infection, Pneumonia, Case report

## Abstract

**Background:**

Tumor necrosis factor-α (TNF-α) antagonists, most of which are monoclonal antibodies, became a widespread treatment for autoimmune diseases such as rheumatoid arthritis, ankylosing spondylitis, inflammatory bowel diseases, psoriasis, psoriatic arthritis, hidradenitis suppurativa and uveitis. Their use is based on the blockage of TNF-α, which plays an important role in granulomas formation, development of phagosomes, activation and differentiation of macrophages, immune response against viral pathogens. The multiple adverse effects of TNF-α inhibition have been identified, including a two-to four-fold increased risk of active tuberculosis and other granulomatous conditions and an increased occurrence of some other serious bacterial, fungal and certain viral infections.

**Case presentation:**

A 34-year-old male patient with disseminated varicella and pneumonitis was admitted to our hospital. The diagnosis of varicella was established serologically by enzyme immunoassay (EIA) and by polymerase chain reaction confirmation of the virus in vesicular fluid. The patient has been receiving adalimumab and methotrexate for the last 3 years due to ankylosing spondylitis and was seropositive to varicella zoster virus prior to the introduction of TNF-α antagonists. Acyclovir was administered for 10 days with the resolution of clinical illness and radiological signs of pneumonitis.

**Conclusion:**

Due to the use of biological agents, particularly TNF-α inhibitors, as a well-established therapy for some autoimmune diseases, new potential adverse events can be expected in the future and we wanted to point out one of them. To our knowledge this is the first case of recurrent disseminated varicella in a patient taking TNF-α antagonists.

## Background

Tumor necrosis factor-α (TNF-α) antagonists, most of which are monoclonal antibodies (infliximab, golimumab, adalimumab), became a widespread treatment for autoimmune diseases such as rheumatoid arthritis, ankylosing spondylitis, inflammatory bowel diseases, psoriasis, psoriatic arthritis, hidradenitis suppurativa and uveitis. Their use is based on the blockage of TNF-α, which plays an important role in granulomas formation, development of phagosomes, activation and differentiation of macrophages, immune response against viral pathogens [1–3]. Adalimumab is a recombinant human immunoglobulin (Ig) G monoclonal antibody specific for human TNF-α which causes modulation of the inflammatory response activated by this cytokine.

However, multiple adverse effects of TNF-α inhibition have been identified, including a two-to four-fold increased risk of active tuberculosis and other granulomatous conditions and an increased occurrence of some other serious bacterial, fungal and certain viral infections [4–6].

## Case presentation

We report a case of a 34-year-old patient with a medical history of fever, malaise, cough, and generalized vesicular rash that started 1 day before admission. 14 days prior to disease onset, the patient’s son developed chickenpox. The patient had a history of ankylosing spondylitis and has been treated with adalimumab 40 mg subcutaneously biweekly in addition to methotrexate10 mg per week for the last 2 years. He had had chickenpox at the age of 5, and positive IgG antibodies (titre 24; positive > 11) to varicella-zoster virus (VZV) using EIA in 2014, prior to the initiation of adalimumab treatment.

**Fig. 1:**
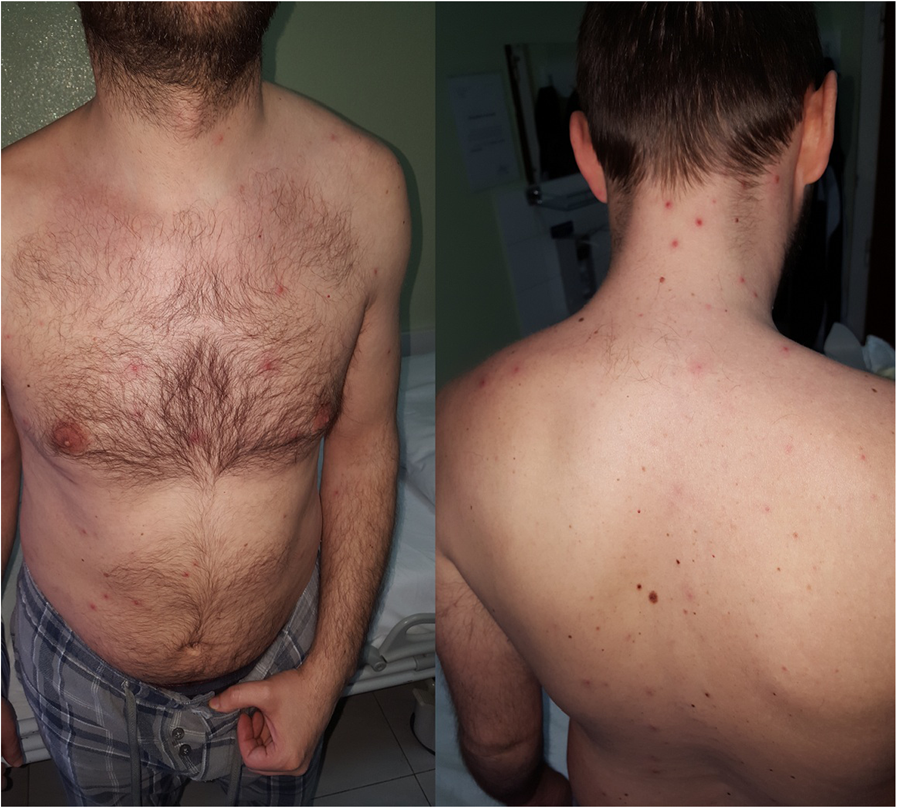
Papular and vesicular rash on the neck and trunk

**Fig. 2:**
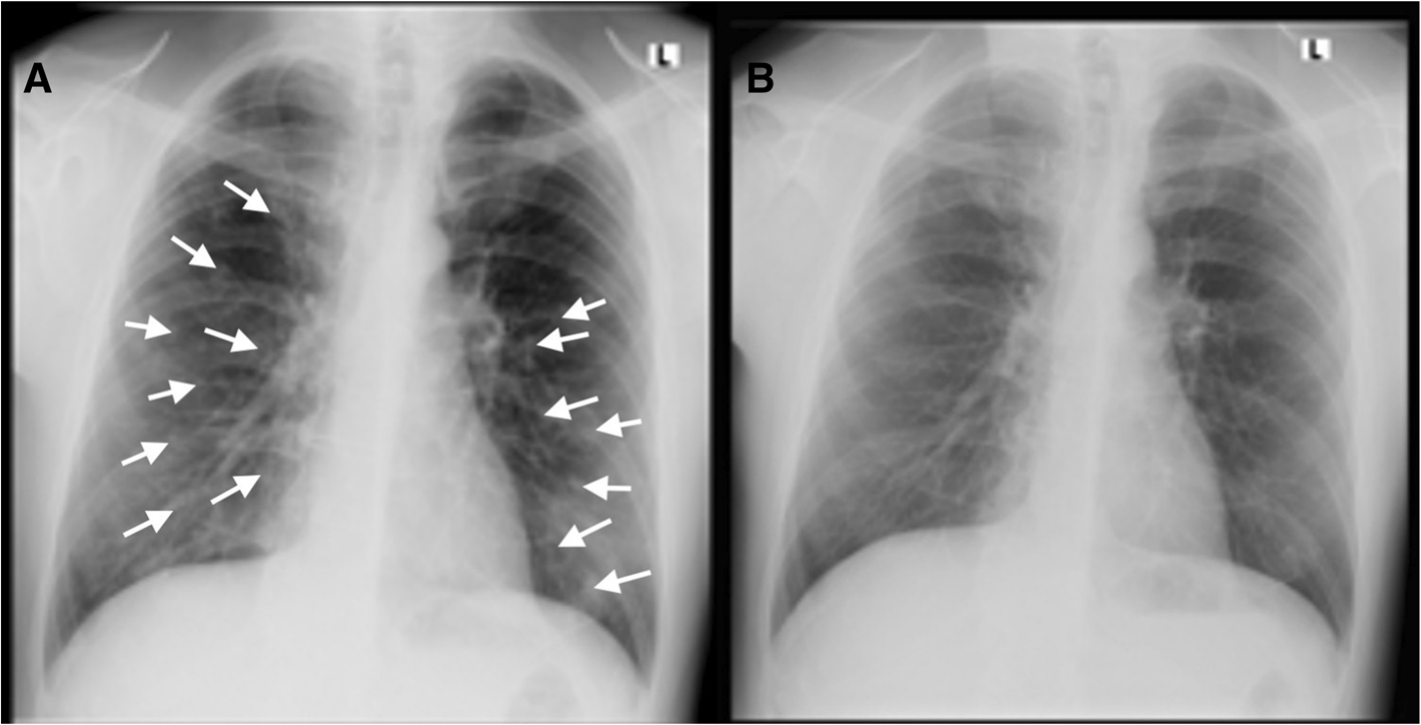
**a** Chest radiograph on the day of admission revealed diffuse nodular infiltrates. **b** Radiological resolution of pulmonary infiltrates after 10 days of acyclovir therapy

On admission to the hospital, physical examination revealed a subfebrile (37.3 °C) patient with papular and vesicular rash over the entire body (Fig. 1). Laboratory test results showed: erythrocyte sedimentation rate 17 mm/1st hour, C-reactive protein 17,7 mg/l, white blood cell count 4,8 × 109/l with 56% neutrophils and 25% lymphocytes in differential count, elevated fibrinogen (3,1 g/l). Other standard parameters such as haemoglobin concentration, platelet count, glucose concentration, plasma ion levels, renal and liver functional tests, coagulation tests and urinalysis were all normal. A chest radiograph showed diffuse bilateral nodular infiltrates (Fig. 2a). Recurrent varicella infection was suspected and intravenous acyclovir was administered (10 mg/kg every 8 h). A Tzanck smear revealed multinucleated giant cells and VZV deoxyribonucleic acid (DNA) was detected in vesicular fluid by polymerase chain reaction. Serological testing for VZV using EIA was performed on the third day of illness and IgM (titre 15; positive > 11), IgG (titre 36; positive > 11) and IgA (titre 12; positive > 11) antibodies to VZV were detected. Based on the clinical and laboratory findings, the diagnosis of recurrent varicella with pneumonia was established. The patient was treated with intravenous acyclovir (750 mg every 8 h) for 7 days, followed by oral acyclovir (800 mg five times daily) for 3 more days. He remained febrile for 3 days with rapid resolution of the rash and radiological resolution of pulmonary infiltrates (Fig. 2b).

## Discussion and conclusions

Patients receiving TNF-α antagonists therapy remain at a selectively increased risk for more severe primary varicella infections compared with the general population, with the estimated incidence rate of hospitalization due to chickenpox of 26 cases per 100,000 (95% CI 10–69) compared with the expected rate of 1.9 (95% CI 1.8–2.0) in the general population [7]. Contradictory results have been published reporting the association between TNF-α therapy and herpes zoster.

Several large population-based studies have been conducted, with those performed in the United States not finding an increased risk with TNF-α inhibitors, contrary to those in Europe generally showing an increased risk [4, 8, 9]. There were multiple reports of severe and disseminated herpes zoster and primary varicella associated with TNF-α therapy (infliximab and adalimumab) [1, 10–15]. In contrast, recurrent varicella infections during TNF-α therapy were rarely reported, with only one case featuring few clinical symptoms (no dissemination and resolution of skin lesions without antiviral treatment) in the course of etanercept administration [16]. VZV causes two clinically distinct diseases - primary infection resulting in varicella and herpes zoster resulting from the reactivation of latent VZV that gained access to sensory ganglia during varicella [17]. It is traditionally considered that VZV infection provides lifelong immunity but recurrent infection (also referred to as reinfection or second varicella infection) with VZV occur more commonly than previously thought [18, 19]. In an active surveillance initiative in California, the percentage of patients diagnosed with varicella who reported previous varicella infections ranged from 4.5 to 13.3% [18]. VZV recurrent infection can occur even in immunocompetent patients, and our patient was predisposed to infection by his rheumatologic disease and immunosuppressive therapy. Patients with a history of underlying malignancy, steroid use or immunosuppressive therapy, HIV infection, or solid organ transplantation are susceptible for disseminated varicella due to impaired cellular immunity. Clinical manifestations in the immunosuppressed host can include atypical and severe manifestations such as development of crops of vesicles over weeks, large and haemorrhagic skin lesions, pneumonia, or widespread disease with disseminated intravascular coagulation [17, 20]. The antiviral therapy is recommended for immunocompromised hosts who present with varicella because they are at risk for developing disseminated varicella, as our patient has been, and can also experience more frequent severe morbidity and higher mortality rates compared with immunocompetent hosts [20, 21]. Early clinical recognition of VZV infection in high risk patients, such are all immunocompromised patients, as well as laboratory detection and confirmation of VZV require early aggressive antiviral treatment leading to favourable clinical outcome [7, 17, 20].

Due to the use of biological agents, particularly TNF-α inhibitors, as a well-established therapy for some autoimmune diseases, new potential adverse events can be expected in the future and we wanted to point out one of them. To the best of our knowledge, this is the first case of confirmed recurrent varicella with disseminated disease and pneumonia in a patient receiving adalimumab since this drug has been approved for use in 2002 in the United States and 2003 in the European Union [22, 23].

## Abbreviations

DNA: Deoxyribonucleic acid
EIA: Enzyme immunoassay test
Ig: Immunoglobulin
TNF-α: tumor necrosis factor-α
VZV: Varicella-zoster virus
